# *Penicillium expansum:* biology, omics, and management tools for a global postharvest pathogen causing blue mould of pome fruit

**DOI:** 10.1111/mpp.12990

**Published:** 2020-09-23

**Authors:** Dianiris Luciano‐Rosario, Nancy P. Keller, Wayne M. Jurick

**Affiliations:** ^1^ Department of Plant Pathology University of Wisconsin at Madison Madison Wisconsin USA; ^2^ Department of Medical Microbiology and Immunology Department of Bacteriology Food Research Institute University of Wisconsin at Madison Madison Wisconsin USA; ^3^ Food Quality Laboratory USDA‐ARS Beltsville Maryland USA

**Keywords:** blue mould, disease management, food loss, genomics, mycotoxins, pome fruit, postharvest decay, virulence regulators

## Abstract

Blue mould, caused primarily by *Penicillium expansum*, is a major threat to the global pome fruit industry, causing multimillion‐dollar losses annually. The blue mould fungus negatively affects fruit quality, thereby reducing fresh fruit consumption, and significantly contributes to food loss. *P. expansum* also produces an array of mycotoxins that are detrimental to human health. Management options are limited and the emergence of fungicide‐resistant *Penicillium* spp. makes disease management difficult, therefore new approaches and tools are needed to combat blue mould in storage. This species profile comprises a comprehensive literature review of this aggressive pathogen associated with pomes (apple, pear, quince), focusing on biology, mechanisms of disease, control, genomics, and the newest developments in disease management.

**Taxonomy:**

*Penicillium expansum* Link 1809. Domain Eukaryota, Kingdom Fungi, Phylum Ascomycota, Subphylum Pezizomycotina, Class Eurotiomycetes, Subclass: Eurotiomycetidae, Order Eurotiales; Family Trichocomaceae, Genus *Penicillium*, Species *expansum*.

**Biology:**

A wide host range necrotrophic postharvest pathogen that requires a wound (e.g., stem pull, punctures, bruises, shoulder cracks) or natural openings (e.g., lenticel, stem end, calyx sinus) to gain ingress and infect.

**Toxins:**

Patulin, citrinin, chaetoglobosins, communesins, roquefortine C, expansolides A and B, ochratoxin A, penitrem A, rubratoxin B, and penicillic acid.

**Host range:**

Primarily apples, European pear, Asian pear, medlar, and quince. Blue mould has also been reported on stone fruits (cherry, plum, peach), small fruits (grape, strawberry, kiwi), and hazel nut.

**Disease symptoms:**

Blue mould initially appears as light tan to dark brown circular lesions with a defined margin between the decayed and healthy tissues. The decayed tissue is soft and watery, and blue‐green spore masses appear on the decayed area, starting at the infection site and radiating outward as the decayed area ages.

**Disease control:**

Preharvest fungicides with postharvest activity and postharvest fungicides are primarily used to control decay. Orchard and packinghouse sanitation methods are also critical components of an integrated pest management strategy.

**Useful websites:**

Penn State Tree Fruit Production Guide (https://extension.psu.edu/forage‐and‐food‐crops/fruit), Washington State Comprehensive Tree Fruit (http://treefruit.wsu.edu/crop‐protection/disease‐management/blue‐mold/), The Apple Rot Doctor (https://waynejurick.wixsite.com/applerotdr), penicillium expansum genome sequences and resources (https://www.ncbi.nlm.nih.gov/genome/browse/#!/eukaryotes/11336/).

## Graphical abstract

This article is a synthesis and compilation of the latest information on the mycotoxingenic blue mould fungus from multiple perspectives that entail omics, biology, and tools for decay control.

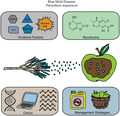

## 1 INTRODUCTION

Apples and processed apple products are worldwide foods consumed by young and old alike that have a farm‐gate value of approximately US$5 billion with downstream revenues of US$15 billion annually (US Apple Association). Postharvest apple diseases (e.g., blue mould) present a significant obstacle to safe access to these foods. Blue mould caused by *Penicillium expansum* and other *Penicillium* spp. is one of the most common global and economically important postharvest fruit rot diseases (Zhong *et al*., 2018). Conservative estimates of blue mould incidence in the United States range from 1% to 5% on fungicide‐treated fruit (author's personal observation). Hence, loses due to blue mould can be estimated to result in US$50–250 million each year. *Penicillium* spp. produce carcinogenic mycotoxins that pose risks to human health when blue mould‐infected fruit are used to make processed products (e.g., juice, sauce, pie filling, fruit butters). Four postharvest fungicides are currently registered for disease management; however, fungicide‐resistant populations of this pathogen have emerged, thus greatly reducing their efficacy (Li and Xiao, 2008; Malandrakis *et al*., 2017). A further complicating factor is the lack of host‐based blue mould resistance to blue mould in commercial apple cultivars. Therefore, innovative approaches are needed to develop new methods to manage this disease. With the advent of the genomic sequence of this pathogen, fundamental research has identified genes and pathways that may lead to mitigation strategies for blue mould decay during storage.

## 2 BLUE MOULD BIOLOGY AND THE POSTHARVEST LIFESTYLE

**FIGURE 1:**
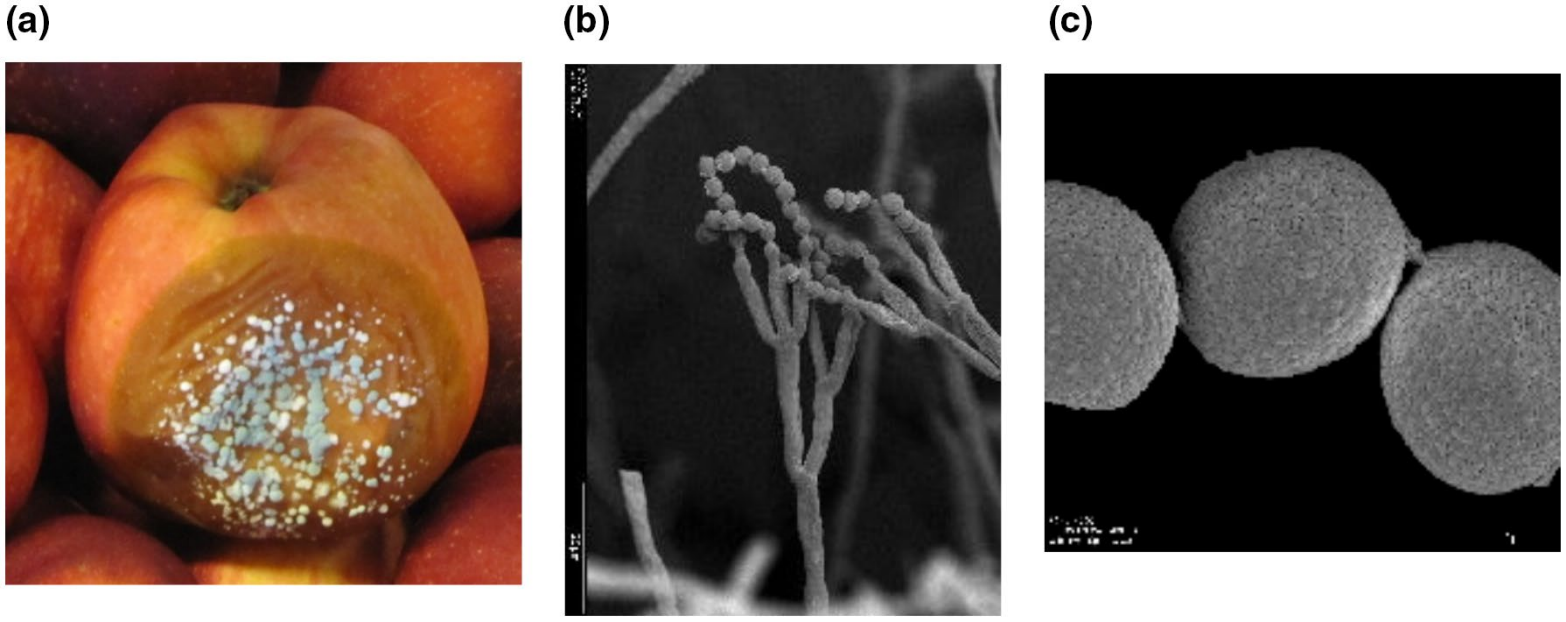
Blue mould decay on apple fruit infected with *Penicillium expansum*. (a) Apple fruit with blue mould symptoms caused by *P. expansum*. (b) Scanning electron micrograph (SEM) of a *P*. *expansum* conidiophore with conidia borne terminally in chains. (c) SEM photograph of *P. expansum* conidia

Apples and pears are stored for extended periods of time to preserve quality and provide fruit throughout the year to meet customer demands (up to 6 months at 1°C in air, and up to 1 year in a controlled atmosphere). There is no host‐based resistance in commercial apple cultivars to fungal plant pathogens that cause postharvest decay, including *Penicillium* spp. (Spotts *et al*., 1999). *Pe* *nicillium expansum* is the most common and economically important postharvest fruit rot pathogen that causes blue mould (Rosenberger, 1990; Xiao and Boal, 2009). While *P. expansum* is the most common and aggressive *Penicillium* species, other *Penicillium* spp., some *Aspergillus* spp., *Byssochlamys* spp., *Colletotrichum* spp., and *Mucor* spp. and other pathogens such as *Botrytis cinerea*, *Botryosphaeria dothidea*, *Botryosphaeria obtusa*, *Alternaria alternata*, and *Neofabraea malicorticis* also cause postharvest rots (Rice *et al*., 1977; Sanderson, 1995; Sholberg and Haag, 1996; Pianzzola *et al*., 2004; Sutton *et al*., 2014). In addition, there is still an array of pome fruit rot pathogens that are being identified and characterized (Khodadadi *et al*., 2020). The disease is of international concern and blue mould fungi are globally distributed. Decay is characterized by a soft, watery rot that is light brown in colour accompanied by the appearance of blue‐green conidia on the fruit surface that develops at advanced stages of decay (Figure 1a). Although some postharvest pathogens can infect fruits directly, *P. expansum* does not as it requires wounds, often caused by stem punctures and severe bruises, that occur before, during, and after harvest. Infection also proceeds via natural openings like lenticels, open calyx/sinus, and stem pull areas (Errampalli, 2004; Rosenberger *et al*., 2006; Wenneker and Thomma, 2020). The fungus produces conidia terminally in chains and primarily reproduces asexually (Figure 1b), although the genome contains two mating types suggestive of a sexual stage (Julca *et al*., 2015). Conidia are the primary source of inoculum and are found in the packinghouse in flume water, on bin surfaces, on fruit, and in the air (Sutton *et al*., 2014).

## 3 VIRULENCE FACTORS

Many research efforts have been focused on identifying genes and gene products that contribute virulence and decay development in *P. expansum*. In this section we present the clearest advances in understanding the processes contributing to *P. expansum* virulence and toxins.

### 3.1 Cell wall‐degrading enzymes

Most necrotrophic fungal pathogens use and deploy an arsenal of cell wall‐degrading enzymes (CWDEs) in order to colonize their respective hosts. Often, these enzymes are functionally redundant. In *P. expansum*, polygalacturonases have been identified as important components in blue mould disease development and are primarily responsible for tissue maceration and pathogen colonization. Polygalacturonase 1 (PG1) was shown to be an important enzyme for disease development in *P. expansum* as it was extracted from decayed tissue exclusively (Yao *et al*., 1996). To determine the regulatory cues that *P. expansum* needs to have optimal polygalacturonase activity, results from in vitro studies suggest that pectin as a carbon source leads to maximum polygalacturonase activity when compared to glucose, galactose, apple, and pear tissues. Interestingly, when growth was assessed in these conditions, apple and pear showed the highest mycelial weight. It is also established there is an inverse correlation between the secretion of polygalacturonases and fungal growth (Jurick *et al*., 2012). The importance of CWDEs in virulence is supported by several RNA‐Seq studies of *P. expansum*‐infected apples where such genes are among those most highly expressed during infections (Tannous *et al*., 2018b; Wang *et al*., 2019).

### 3.2 PacC: a pH regulator involved in acidifying host tissue

The paradigm that many plant pathogens alter the host pH resulting in enhanced virulence is widely studied and accepted (Alkan *et al*., 2013). While some pathogens generate and thrive in alkaline conditions, others like *P. expansum* acidify the host tissue to enhance colonization. A series of studies have focused on understanding how *P. expansum* lowers the host's pH through production of gluconic, citric, and fumaric acids (Prusky *et al*., 2004; Alkan *et al*., 2013), findings more recently supported by transcriptomic data (Barad *et al*., 2016b). A clear picture has emerged that this process is mediated by PacC, a conserved transcription factor involved in pH regulation in many fungi (Selvig and Alspaugh, 2011). Chen *et al*. showed that *pacC* is up‐regulated at alkaline conditions with a maximum expression at pH 9 and cleaved to become active at pH 5. This protein is essential for virulence, growth, and conidiation as results showed reduced outcomes in Δ*pacC* when compared to the wild‐type (WT) strain (Chen *et al*., 2018). To explore the mechanism of PacC regulation, two genes that are directly regulated by PacC were identified and assessed for their contribution to virulence. These genes, *PeSat* (sulfate adenylyltransferase) and *PeGod* (glucose oxidase, *Gox2*), contributed to virulence, although to a reduced extent (Chen *et al*., 2018). Currently, we understand that host pH decrease is triggered by *P. expansum* as follows: (a) alkaline pH due to ammonia produced at the leading edge of the infection activates the PacC transcription factor, which is subsequently cleaved to its active form (Barad *et al*., 2016a; Chen *et al*., 2018), and (b) the active form of PacC positively regulates glucose oxidase (*Gox2*) expression, which leads to the conversion of glucose to gluconic acid, resulting in host tissue acidification and optimal activity of CWDEs like PG1 (Prusky *et al*., 2004; Hadas *et al*., 2007; Barad *et al*., 2012; Chen *et al*., 2018).

### 3.3 LaeA

LaeA (Loss of AflR Expression) is a global regulator of secondary metabolism and a virulence factor in many filamentous fungal species (Bok and Keller, 2016). In *P. expansum*, LaeA was studied by generating deletion mutants (Δ*laeA*) in two *P. expansum* strains: Pe21, an isolate from Israel, and T01, an isolate from China (Kumar *et al*., 2016). LaeA was shown to be a positive regulator of patulin in both isolates as decreased amounts of the mycotoxin were detected in apples inoculated with Δ*l* *aeA* strains when compared to those inoculated with the WT strains. Apples inoculated with Δ*laeA* also showed reduced decay when compared to apples infected with the WT strain in both backgrounds. The extent of this reduction was variable depending on the fungal strain. For instance, the T01 Δ*laeA* mutant did not show any virulence defect in early harvested fruits but only in late harvested fruits, while the Pe21 Δ*laeA* mutant was less virulent than WT in both fruit stages. The variation of *laeA* loss in the two genetic backgrounds reflects an emerging understanding of heterogeneity in pathogenic species and confounds the use of any one reference strain in virulence studies (Keller, 2017).

### 3.4 VeA

VeA, named after its velvet domain, is part of the heterodimeric velvet complex that regulates fungal development and secondary metabolism (Sarikaya‐Bayram *et al*., 2015). In *P. expansum*, *veA* was shown to impact fungal development and secondary metabolism, as Δ*veA* strains showed a conidiophore defect in vitro and no patulin or citrinin production when compared to the WT strain (El Hajj Assaf *et al*., 2018). In addition, *veA* was shown to be involved in virulence as a reduced growth diameter after inoculations in apples was observed for the Δ*veA* strain when compared to the WT, along with reduced sporulation and volume of the rot.

### 3.5 CreA

CreA (Carbon Responsible Element) is another highly conserved transcription factor that regulates carbon catabolite repression (CCR) activity in filamentous fungi. Its role in virulence has been described in several fungal species (Luo *et al*., 2014; Beattie *et al*., 2017; Fasoyin *et al*., 2018) and it is a major virulence factor in *P. expansum*, where Δ*creA* mutants are nearly avirulent (Tannous *et al*., 2018b). Results from the previous study on LaeA (Kumar *et al*., 2016) had suggested CreA may negatively regulate LaeA as increasing sucrose concentrations resulted in decreased *laeA* expression, decreased patulin synthesis, but increased *creA* expression (Kumar *et al*., 2016). To investigate if this was the regulatory mechanism of patulin production, *creA* deletion (Δ*creA*) strains were generated and compared to WT (Tannous *et al*., 2018b). Δ*creA* mutants showed reduced colony diameter and conidial production, delayed germination, and reduced patulin production when grown on different carbon sources. Patulin production in vivo was also significantly reduced in the Δ*creA* strains. While patulin production decreased in the Δ*creA* in vitro, interestingly this strain showed the genes for the patulin biosynthetic cluster to be significantly up‐regulated when compared to the WT strain, suggesting patulin regulation occurs at a posttranscriptional level. Thus, while it had been previously hypothesized that CreA regulated patulin production via down‐regulation of patulin genes via LaeA, these results suggested an alternative CreA‐mediated regulatory mechanism(s).

### 3.6 SntB

SntB, described initially for its Sant domain, is an epigenetic reader protein regulating secondary metabolism that was recently described in *Aspergillus nidulans* and *Aspergillus flavus* (Pfannenstiel *et al*., 2017, 2018). In *A. flavus* the Δ*sntB* mutant showed decreased colonization and conidial production when compared to the WT strain upon inoculation of maize kernels (Pfannenstiel *et al*., 2018). In muskmelon, a reduction in virulence was also reported showing *Fusarium oxysporum* f. sp*. melonis* Δ*snt2* causing reduced plant mortality when compared to the WT strain. Deletion of *sntB* in *P. expansum* also resulted in a less virulent strain (Tannous *et al*., 2020). Δ*sntB* strains of *P. expansum* were reduced in conidiation and colony diameter compared to WT in vitro and also showed smaller lesion development in apple. Patulin synthesis was significantly decreased both in vitro and in vivo in the Δ*sntB* when compared to the WT.

### 3.7 PeLysM1, PeLysM2, PeLysM3, and PeLysM4

Fungal LysM (Lysin motif) domain‐containing proteins have been described in an array of phytopathogens and were originally discovered in *Cladosporium fulvum* (de Jonge *et al*., 2010). These proteins have been found to have different roles, such as protection from host hydrolytic enzymes, and preventing activation of pattern‐triggered immunity (PTI) by scavenging chitin fragments and thus escaping host recognition (Kombrink and Thomma, 2013). The *P. expansum* genome contains 18 LysM containing proteins, 11 of which contain a signal peptide and four of which are expressed during the infection process (Levin *et al*., 2017). In a study aiming to evaluate the role of a subset of these in *P. expansum* virulence, the authors generated single deletion mutants for the genes *PeLysM1*, *PeLysM2*, *PeLysM3*, and *PeLysM4* and found that these do not show reduced lesion diameter on apples when compared to the WT strain (Levin *et al*., 2017).

### 3.8 NLP1 and NLP2 effector proteins

Necrosis and ethylene‐inducing peptide 1 (NEP1)‐like proteins (NLP proteins) or effectors have been shown to be important for virulence in a variety of phytopathogens (Kleeman *et al*., 2012; Feng *et al*., 2014). A study by Levin *et al*. showed that there are two NLP‐encoding genes in the *P. expansum* genome, identified as *PeNLP1* and *PeNLP2* and classified as type I and III NLP effectors, respectively. Transient expression of these proteins in *Nicotiana benthamiana* resulted in a necrosis phenotype, suggesting their involvement in cell death‐mediated processes or in activation of plant defence mechanisms. Expression of *PeNLP1* was up‐regulated through time course studies during apple infection while *PeNLP2* was highly expressed in the spores. Apple inoculation experiments using single gene deletion mutants showed a significant reduction of lesion diameter for the Δ*penlp1* mutant but not the Δ*penlp2* mutant when compared to the WT strain (Levin *et al*., 2019).

### 3.9 Subtilisin‐related peptidase S8‐PePRT

PePRT is a putative subtilisin‐related peptidase identified by a *P. expansum* custom effector prediction pipeline developed in the same study (Levin *et al*., 2019). The application of this pipeline resulted in identifying 17 candidate effector‐encoding genes where *PePRT* was the highest gene in the priority rank list. It is not clear what the function of the *PePRT* gene product is, although its similarity to subtilisin‐related peptidases suggests it may be involved in virulence through autophagy processes and recycling of cellular materials. Gene deletion of *PePRT* showed developmental defects that include reduced colony diameter in vitro, aberrant microscopic hyphal morphology, and a reduction in conidiation when compared to the WT strain. Reduced lesion diameter by the Δ*peprt* strain on apple when compared to WT showed a *PePRT* contribution to the infection process and validated the designed pipeline as an effective tool to identify potential genes that mediate *P. expansum* virulence.

### 3.10 Blistering1

Blistering1 is a DNAJ domain‐containing protein, predicted to be targeted to the mitochondrion, which was identified through a T‐DNA mutagenesis screening performed to uncover genes that contribute to *P. expansum* virulence (Jurick II *et al*., 2020). This gene only has one copy in the *P. expansum* genome and was named blistering1 due to scanning electron micrographs that show “blisters” in the hyphal surface of the T‐DNA mutant instead of the smooth surface phenotype from the WT strain. In vivo apple inoculations show that disruption of this gene led to reduced lesion diameter when compared to the WT strain. Decreased patulin production in vitro was recorded, as well as protein secretion being altered, including the secretion of CWDE in the T‐DNA mutant. Hence, this locus represents the first master regulator of protein secretion in this fungus, for which it was shown that multiple processes for virulence (e.g., patulin, CWDE secretion, pH regulation) were all affected by this single gene.

### 3.11 Mycotoxins

*P. expansum* isolates produce several secondary metabolites, some of which show cellular damage and are considered possible mycotoxins, including chaetoglobosins, citrinin, communesins, expansolides A and B, ochratoxin A, patulin, penitrem A, roquefortine C, and rubratoxin B (Andersen *et al*., 2004; Tannous *et al*., 2018a). However, only two of these metabolites, patulin and citrinin, are widely recognized as mycotoxins of concern and will be briefly described here.

#### 3.11.1 Patulin

Patulin is an β‐unsaturated heterocyclic lactone produced by at least 60 fungal species (Loi *et al*., 2017). The compound is problematic for processed fruit products as it survives pasteurization, is very heat stable, and is not completely eliminated during fermentation during cider production. Originally studied and evaluated as a potential antibiotic, further studies described it as a mycotoxin due to its hepatoxic, genotoxic, and immunotoxic characteristics (Saleh and Gotepe, 2019; Vidal *et al*., 2019). Amongst all these toxins, patulin has been the best characterized and has a strong affinity for sulfhydryl groups that inhibits many mammalian enzymes. Its acute toxicity was demonstrated in rodents with oral LD_50_ ranging between 29 and 55 mg/kg body weight. Notable effects of toxicity include agitation, dyspnea, pulmonary congestion, oedema, ulceration, hyperaemia, and distension of the gastrointestinal tract (Puel *et al*., 2010). Signs of subacute toxicity include neurotoxicity (tremors, convulsions) as well as inhibition of several intestinal enzymes (Devaraj *et al*., 1986; Puel *et al*., 2010). Patulin genotoxicity, teratogenicity, mutagenicity, and immunotoxicity are implied, but inconclusive (Puel *et al*., 2010). Due to its toxic and potentially carcinogenic effects, in 2003 the European Union limited the maximum amount of patulin to 50 μg/L for fruit juices and derived products, 25 μg/L for solid apple products, and 10 μg/L for juices and foods for babies and young infants. The U.S. Food and Drug Administration (FDA), as well as other countries, have imposed strict limits on permissible amounts of patulin in fruit juices and processed pome fruit products for human consumption at 50 µg/L (Puel *et al*., 2010; Zhong *et al*., 2018).

*P. expansum* contains a complete patulin gene cluster consisting of 15 genes that encode 10 biosynthetic enzymes (*patL* [transcription factor], *patK* [6‐methylsalicylic acid synthase], *patG* [6‐methylsalicylic acid decarboxylase], *patH* [*m*‐cresol methyl hydroxylase], *patI* [*m*‐hydroxybenzyl alcohol hydroxylase], *patO* [isoamyl alcohol oxidase]/*pat* *J* [unknown], *patN* [isoepoxydon dehydrogenase], *patF* [unknown], *patD* [alcohol dehydrogenase], and *patE* [glucose–methanol–choline oxidoreductase family protein]) required for synthesis, which is completed in 10 steps (Tannous *et al*., 2014; Ballester *et al*., 2015; Li *et al*., 2015, 2019). While these papers described the patulin cluster composition and organization, a major breakthrough in understanding the contribution of each gene in the biosynthetic pathway was made by Li *et al*. (2019). In this article, the authors confirmed the involvement of all of the genes in the cluster by using gene deletion mutants, substrate feeding experiments, and identifying subcellular localization with tagged fusion proteins. This study shows most enzymes localize to the cytosol (PatB, PatD, PatF, PatG, PatK, PatN), others to the nucleus (PatL), cell periphery and cell wall (PatE), endoplasmic reticulum (ER) (PatA, PatH, PatI), cell membrane (PatC, PatM), vacuoles (PatO), and intracellular vesicles (PatJ) and established that correct subcellular localization is essential for patulin synthesis (Li *et al*., 2019). Patulin is not required for *P. expansum* infection although conflicting results suggest it can contribute to virulence in a cultivar‐dependent manner (Sanzani *et al*., 2012; Barad *et al*., 2014; Ballester *et al*., 2015; Li *et al*., 2015; Snini *et al*., 2016). For instance, Snini *et al*. (2016) showed it is important in seven apple varieties out of 12 tested.

#### 3.11.2 Citrinin

Citrinin is a polyketide synthesized by several *Penicillium, Aspergillus*, and *Monascus* spp. (reviewed in Doughari, 2015). Although citrinin contamination is not monitored by regulatory agencies, its toxicology is known and studies indicate worldwide exposure to citrinin warrant a tougher regulatory stance on this toxin (Ali and Degen, 2019). Citrinin has been shown to have cytotoxic properties for *Schizosaccharomyces pombe* and an array of cell lines in which the effects vary but result in a variety of phenotypes, including inhibition of cell proliferation and triggering apoptosis (Doughari, 2015; de Oleira Filho *et al*., 2017). Citrinin is mainly described as a nephrotoxic although it is also proposed to have tetratogenic effects. This polyketide acts as a nephrotoxic compound in which the mechanism described is RNA synthesis disruption in the kidneys. Other toxicological effects, such as its effect as a skin irritant and carcinogenic potential, have been studied (Doughari, 2015). The LD_50_ for citrinin varies between 80.5 μg/egg in chicken embryo, 57 mg/kg for ducks, and 134 mg/kg in rabbits (Ciegler *et al*., 1977; Doughari, 2015).

Citrinin was originally isolated from *Penicillium citrinum* in 1831 and has since been found to be produced by dozens of Eurotiales fungi (Xu *et al*., 2006; Li *et al*., 2017; de Oleira Filho *et al*., 2017). Citrinin biosynthesis was successfully analysed by heterologous expression of the *Monascus ruber* citrinin gene cluster in *Aspergillus oryzae*. This study showed that five genes from the predicted nine‐gene cluster were sufficient to synthesize the toxin (He and Cox, 2016). However, the citrinin cluster in *P. expansum* is predicted to contain nine genes as opposed to the clusters described for *Monascus aurantiacus* and *Monascus purpureus*, which contain 16 and 6, respectively (Ballester *et al*., 2015). The role and regulation of citrinin in the context of disease progress have not been extensively investigated, although the studies that have been performed present contradictory data. Ballester *et al*. (2015) generated a *P. expansum* Δ*pksCT* strain that was unable to produce citrinin and showed no significant difference when compared to the WT strain in apple inoculation studies. Another study suggests that citrinin is mostly produced at later stages of infection and shows a reduced lesion diameter on apple inoculation with Δ*pksCT* when compared to WT. The authors also present the hypothesis that citrinin acts as an antioxidant to protect mycelia from the host response oxidative environment, specifically reactive oxygen species production (Touhami *et al*., 2018).

## 4 BLUE MOULD GENETICS

### 4.1 Genomics

*P. expansum* strain R19 was the first blue mould fungus to have its genome sequenced (Yu *et al*., 2014). Yang *et al*. (2014) released the *P. aurentiogriseum* NRRL 62431 genome, which was isolated from hazel nut in Oregon. Because this isolate was misidentified and originally published as *P. aurentiogriseum*, later genome‐scale comparisons revealed that NRRL 62431 was *P*.*expansum* and subsequently it was renamed. A total of nine genomes for eight *P. expansum* strains isolated from different geographical areas are available in the NCBI database (Table 1) (Yang *et al*., 2014; Yu *et al*., 2014; Ballester *et al*., 2015; Li *et al*., 2015; Wu *et al*., 2019). Unlike in many other pathosystems, the scientific community that researches *P. expansum* have not adopted a strain as its “standard” to conduct molecular studies on. In general, genomic analyses show the *P. expansum* genomes range between 31.0 and 35 Mbp in size, 10,560 and 11,770 predicted protein‐coding genes, and 53 to 58 predicted secondary metabolite gene clusters (Ballester *et al*., 2015; Li *et al*., 2015; Wu *et al*., 2019). One of the first genomic characterizations revealed the predicted patulin cluster, confirming its initial finding made through RACE‐PCR (Li *et al*., 2015; Tannous *et al*., 2014). In the article by Li *et al*. (2015), the author presents and characterizes the *P. expansum* T01 genome. Ballester *et al*. conducted an in‐depth analysis comparing three geographically distinct *P. expansum* isolates Pe21 (Israel), CMP‐1 (Spain), and MD‐8 (USA). The authors reveal interspecific variation and, as an example,present patulin production levels that differ between the strains. The MD‐8 strain produces a diminished amount of patulin when compared to Pe21 and CMP‐1. Important findings regarding the *P. expansum* sexual stage have also been made through genomics. Julca *et al*. (2015) identified mating types MAT1‐1 for strains Pe21, MD‐8, CMP‐1, NRRL 62431, and R19, while strains ATCC 24692 and T01 carry MAT1‐2 (Julca *et al*., 2015). As next‐generation sequencing technologies continue to have better resolution, questions regarding the variability in disease virulence and its possible correlation to genomes may be more possible to answer. To date, *P. expansum* genomic assemblies are limited to contigs. Recently, a comparative genomics study focused on assessing differences between *P. expansum* and other *Penicillium* species, including the blue mould‐causing *P. solitum*, to identify potential genes that contribute to variable virulence (Wu *et al*., 2019). For the *P. expansum* R19 genome it represents the highest quality assembly to date, with 16 contigs (Wu *et al*., 2019). Functional analyses of specific genes must be performed to validate the predicted role of genomics‐based data.

**TABLE 1:** *Penicillium expansum* genomic resources

*P. expansum* strains	d1 (Pe21/Pe1)	CMP‐1	MD‐8	NRRL 62431 (*P. aurantiogriseum*)^a^	ATCC 24692 (JGI strain)	R19 (2014)	R19 (2019)	T01	R21	YT02
Annotation code	PEXP	PEX1	PEX2	B276	Penex1 (not on NCBI)	U726	EAS63	PEG	BFT88	PEYT02
Sequencing platform	Illumina HiSeq	Illumina HiSeq	Illumina HiSeq	Illimina GaIIX	n/a	Illumina	PacBio	Illumina	Illumina Miseq	454; Illumina GAIIx; Illumina HiSeq
Total sequence length (bp)	32,065,046	31,087,040	32,356,048	31,547,516	32,450,000	31,415,732	32,896,497	33,032,596	35,046,070	31,251,972
Assembly level	270 contigs	1,723 contigs	382 contigs	4,775 contigs	Scaffold	1,231 contigs	16 contigs	Contig	257 scaffolds	Scaffold
Protein‐coding genes	11,023	10,663	11,060	11,476	n/a	10,554	n/a	11,770	n/a	n/a
Accession location	Israel	Spain	United States	United States	n/a	United States	United States	China	United States	China
Isolated from	Apple	Apple	Apple	Hazelnut (*Corylus avellana*)	n/a	Apple cv. Red Delicious	Apple cv. Red Delicious	Apple	Apple‐Red Delicious	n/a
BioSample^b^	SAMN02928572	SAMN02928571	SAMN02928573	SAMN01091924	n/a	SAMN02716838	SAMN10256288	SAMN02385220	SAMN05582703	SAMN03276233
BioProject^b^	PRJNA255745	PRJNA255744	PRJNA255747	PRJNA170336	n/a^c^	PRJNA225688	PRJNA497398	PRJNA222879	PRJNA339168	PRJNA50009

n/a

^a^ NRRL62431 is a *Penicillium expansum* strain originally misidentified as *Penicillium aurantiogriseum*.

^b^ BioSample and Bioproject are NCBI databases. Identifiers are listed in these rows.

^c^ n/a = not applicable.

### 4.2 Transcriptomics

Numerous data sets have been generated for the *P. expansum* pathosystem with the common aim of identifying key genes that contribute to the success of this pathogen. In total, 10 transcriptomic data sets of *P. expansum* are available and these are summarized in Table 2; several of these were obtained from colonized apples and are the ones discussed here. The first transcriptomic data set available included data from *P. expansum* CMP‐1 in 7‐day potato dextrose agar spores and inoculated Golden Delicious apples at 24, 48, and 72 hr postinoculation (hpi) (Ballester *et al*., 2015). The authors could map 6,117 transcripts to the genome predicted to contain 10,683 genes. The authors reported an increased representation of transcripts for exosome, ribosome biogenesis, CWDEs, proteases, and oxidoreductases at all of the time points tested. Interestingly, they identified 20 biosynthetic gene clusters (BGCs) and two glucose oxidases, gox2 and gox3, that were expressed during infection, confirming the results by Barad *et al*. (2012). A recent study expanded the findings of this data set as it includes earlier time points in the infection process (Wang *et al*., 2019). A *P. expansum* isolate was used to inoculate apple and extract RNA at 0, 1, 3, and 6 hpi as early transcript events were thought to be most useful to predict genes that contribute to virulence (Wang *et al*., 2019). In a previously mentioned study by Tannous *et al*. (2018b), a comparative transcriptomics data set was obtained from WT and Δ*creA* strains inoculated in Golden Delicious apples 5 days postinoculation. Because the Δ*creA* strain is nearly avirulent, this data set provides an opportunity of generating more hypotheses or gene targets that may contribute to virulence and be targeted for further characterization. In this study, 399 genes were significantly differentially expressed between the WT and Δ*creA* strains. Of these, 244 genes were up‐regulated and 155 down‐regulated in the Δ*creA* strain when compared to the WT. Among the up‐regulated transcripts in the Δ*creA* strain, the authors report transcripts for the patulin and citrinin cluster, thus supporting the hypothesis that this product is regulated posttranscriptionally in this strain. In another study, with the aim of identifying pH‐regulated genes in vivo, Barad *et al*. (2016b) reported the transcriptomes of *P. expansum* grown in (a) culture medium at pH 4, and (b) culture medium at pH 7. For these treatments, the samples were taken at different time points (0.5, 1, 3, 10, and 24 hpi). They also included a sample from the leading edge of colonized apple tissue. For these data sets, 5,646 genes were significantly differentially expressed among all the treatments. With this approach, the authors could identify genes and processes that are potentially important for apple colonization and are possibly pH regulated, and vice versa.

**TABLE 2:** Summary table of *Penicillium expansum* transcriptomic studies

Objective	Assessed conditions	Summary	Transcripts	Strain used	GO categories	Technology	Reference
Identify expressed genes in the infection process	RNA was assessed from infected apples at 24, 48, and 72 hpi. The samples were normalized to RNA from spores grown on potato dextrose agar	Reported expression of 20 putative metabolic clusters. At 24 hpi ribosome, exosome protein phosphatases, ubiquitin system, DNA repair, and recombination proteins were up‐regulated at least 2 fold. Up‐regulation of proteases, CWDE, oxidoreductases, pectate lyase, and polygalactorunase, two glucose oxidase genes.	384,181 total reads and 6,117 mapped transcripts out of 10,683 predicted genes	CMP‐1	n/a	Roche 454 FLX Titanium System	Ballester *et al*. (2015)
Identify transcripts involved in germination	Samples consisted of tissue 6 hpi and 12 hpi in potato dextrose broth shaking	Most of the DEGs are in the KEGG categories of metabolism and genetic information processing categories. This study reported 77 transcription factors were differentially expressed.	23,950 total reads an reported 3,026 DEGs	Local isolate from an infected apple from a market in Zhejiang, China	Biological process: cellular, metabolic regulation process Cellular component: cell, cell part, organelle; molecular function:binding, catalytic activity, structure molecule activity	BGISEQ‐500	Zhou *et al*. (2018a)
Identify DEGs between wild type and Δ*creA* (near‐avirulent) strains	Inoculated apples with wild type or Δ*creA* (5 hpi)	Up‐regulated in Δ*creA* carbon utilization, glucogenesis, patulin, and citrinin cluster, transporters Down‐regulated in Δ*creA* are glucose transporters, carbohydrate catabolism, concanavalin lectin glucanases, amino acid degradation	399 DEGs, 244 up‐regulated, 155 down‐regulated	Pe21	Single organism metabolic process, oxidoreductase activity, iron ion binding (top three up‐regulated only significant)	Illumina HiSeq 2500	Tannous *et al*. (2018b)
Identify DEGs under patulin permissive conditions (static culture) and patulin restrictive conditions (shaking culture)	CY liquid medium shaking and static cultures 4 days	Up‐regulated in static conditions: LaeA, patulin cluster, ribosome pathway (KEGG). Differential expression of CYP450s, ABC and MFS transporters, SM backbone genes (23 up‐regulated and 14 down‐regulated in static conditions)	3,034 DEGs, 1,642 up‐regulated in static conditions and 1,392 down‐regulated	T01	Catalytic activity, metabolic processes	Illumina HiSeq2000	Li *et al*. (2015)
Identify genes in important pH‐response pathways that mediate apple colonization	Liquid cultures at pH 4, pH 3 at 0.5, 1, 3. 10, 24 hpi and leading edge of infection	Classified DEGs in nine distinct coexpressed gene cluster categories based on expression patterns from the samples: (a) colonized tissue was different to culture conditions, (b) colonized tissue was similar to pH 4, (c) colonized tissue was similar to pH 7	5,646 genes were significantly differentially expressed	Pe21	Up‐regulated at pH 4 in apple and culture but not at pH 7. 67 transcripts 15 GO categories, including nucleobase transport, integral component of membrane, taurine metabolic process. Growth in alkaline conditions 482 genes up‐regulated, 19 GO categories pectate lyase activity, metal ion binding regulation of pH, disaccharide metabolic process	Illumina HiSeq2500	Barad *et al*. (2016b)
Identify genes expressed in early steps of apple infection	Inoculated apples at 1, 3, and 6 hpi	Decreasing trend over time: tryptophan metabolism, valine leucine and isoleucine degradation, tyrosine metabolism, fatty acid degradation, peroxisome. Increasing trend over time: cyanoamino acid metabolism, base excision repair, homologous recombination, mismatch repair, DNA replication.	A total of 386 (1 hpi), 3,387 (3 hpi), and 4,464 (6 hpi) DEGs. 1 hpi, 200 up/ 186 down 3 hpi 1,528 up/ 1839 down 6 hpi 2,306 up/2,158 down	n/a	CWDE, antioxidative stress, pH regulation, effectors	Illumina HiSeq X Ten	Wang *et al*. (2019)
Compare gene expression on samples exposed and not exposed to decanal	Potato dextrose broth with or without decanal at 6, 12, and 18 hpi	As time increased categories in RNA processing, ribosome metabolism and amino acid metabolism increased in decanal‐treated samples. Also oxidative phosphorylation and ATPases genes were down‐regulated under decanal stress. 138 transcription factors differentially expressed under decanal stress. KEGG categories for translation were recorded.	Transcripts were mapped to 74.23% of the genome. DEGs 2,362 (6 hr), 3,249 (12 hr), and 2,963 (18 hr) between decanal treated and nontreated	Local isolate from an infected apple from a market in Zhejiang, China	GO categories biological process: cellular component organization or biogenesis, metabolic process, single cell organism process. Cellular component: cell, cell part, membrane organelle. Molecular function: catalytic activity.	BGISEQ‐500	Zhou *et al*. (2018b)
Compare gene expression on samples exposed and not exposed to cinnamaldehyde and citral	Potato dextrose broth with or without 0.5×MIC of cinnamaldehyde and citral 5 dpi at 25°C	DEGs involved in secondary metabolism, amino acid metabolism and redox processes	A total of 1713 DEGs: 793 down‐regulated, 920 up‐regulated	F‐WY‐12‐02	n/a	Illumina HiSeq2000	Wang *et al*. (2018)
Evaluate the transcriptome of solid‐state fermentation and submerged fermentation with an emphasis on secondary metabolism	0, 6, and 14 day cultures from solid‐state fermentation and submerged fermentation	A total of 79 genes of the significantly expressed transcripts encoded for secondary metabolism genes	901 genes were significantly expressed between the two treatments	KKACC 40815 from the Korean Agricultural Culture Collection	n/a	Illumina HiSeq2500	Kim *et al*. (2016)

DEGs, differentially expressed genes. CWDE, cell wall‐degrading enzyme. KEGG, Kyoto Encylcopedia of Genes and Genomes. hpi, hr postinoculation. MIC, minimum inhibitory concentration.

n/a = not applicable.

## 5 CONVENTIONAL POSTHARVEST DECAY MANAGEMENT STRATEGIES

### 5.1 Chemical and biological controls

Long‐term storage, coupled with lack of host resistance in apple and pear, provides limited options for the industry but to rely on synthetic fungicides to manage postharvest decays (Rosenberger, 2012). Postharvest fungicide applications vary, depending on the stage of postharvest handling, and are typically applied in bin drenches before storage, sprays on sorting lines, dips, application with waxes, or thermofogged in storage rooms. There are four fungicides (Academy, Scholar, Penbotec, and Mertect) registered and being used in the USA for pome fruits to manage postharvest blue mould decay caused by *Penicillium* spp. Both Scholar (active ingredient fludioxonil) and Penbotec (active ingredient pyrimethanil) were labelled for postharvest use in 2004 (Xiao and Boal, 2009). The newest material to be released (Academy, introduced in 2016) contains two single‐site mode of action ingredients, fludioxonil and difenoconazole, to manage blue mould on pome fruits in storage.

Advances using biological controls have been made to combat postharvest rots at the commercial level with the development of BioSave, which contains a saprophytic strain of *Pseudomonas syringae* originally isolated from the surface of apple fruit. It was introduced in 1996 and labelled in the United States to control postharvest diseases of fruits and vegetables, including blue and grey mould on pome and stone fruits (Janisiewicz and Jeffers, 1997).

### 5.2 Fungicide resistance management

Fungicide resistance monitoring is critical to maintain the efficacy of materials with single‐site mode of action that are prone to develop resistance, which is based on developing tools (both conventional in vitro and molecular‐based methods) to detect fungicide‐resistant pathogen populations. Routine monitoring of EC_50_ values and minimum inhibitory concentrations (MICs) can detect shifts in baseline sensitivities that may indicate the development of fungicide resistance that results in decay. Baseline information is derived from a representative pathogen population before an active ingredient is introduced to market (Russell, 2004). This allows researchers to establish an MIC or discriminatory dose for a specific active ingredient generated from an unexposed pathogen population based on EC_50_ values. A recent study by Jurick II *et al*. (2019) determined the baseline sensitivity of difenconazole for *Penicillium* spp. obtained from apple‐producing areas. Their study showed that fungicide‐resistant isolates can be controlled by Academy and a specific dose of difenoconazole to be used to monitor shifts in sensitivity in blue mould populations.

### 5.3 Cultural practices and sanitation

Postharvest decay caused by fungal plant pathogens can be managed more effectively by careful harvest and handling practices that decrease fungal inoculum (spores, conidia, and other survival/resting structures) levels. Good cultural practices in the orchard should focus on the removal of organic debris and decayed fruits. After harvest, bin sterilization, stringent packinghouse sanitation, packing dry fruit, culling bruised and infected fruit, and the maintenance of proper storage temperature are all essential for managing postharvest decay (Rosenberger, 2012). Specific sanitation methods for packinghouses include treating bins with steam, cleaning packinghouse walls and floors with quaternary amines or peroxyacetic acids, and maintaining proper chlorine levels in sizing flumes (Spotts and Cervantes, 1994; Rosenberger, 2012). In addition to being effective on their own merits, cultural practices function synergistically when integrated with either chemical or biological control methods, which increases their efficacy. Thus, they should be practised as part of an integrated control strategy to achieve the highest level of postharvest decay management to ensure treatment efficacy while reducing the risk of developing fungicide‐resistant pathogen populations.

### 5.4 Identifying resistance in wild apples to generate rot‐resistant cultivars

Commercial fruit cultivars are devoid of resistance to postharvest decay caused by fungal plant pathogens (Janisiewicz *et al*., 2008; Jurick II *et al*., 2011). This is especially the case in apple fruit as postharvest disease resistance has not been routinely considered an important part of apple breeding programmes worldwide. However, the focus has been on field diseases, production, flavour, and so on, and not toward resistance to postharvest decay (Janisiewicz *et al*., 2008). Because host‐based resistance is an important part of an integrated disease management strategy, it is important to understand the fundamental mechanisms and identify the genes and networks controlling resistance and incorporate them into agricultural commodities. Central Asia is where apples originated and wild apple forests remain to this day in Kazakhstan (Hokanson *et al*., 1997). In the 1990s, multiple collecting trips for wild apple germplasm were conducted by the U.S. Department of Agriculture, which resulted in the establishment of the Kazakh wild apple germplasm collection in Geneva, New York (Forsline *et al*., 2003). This collection includes a wide variety of phenotypes and genetic traits, and represents a wide array of genetic diversity. It has been the cornerstone of fundamental and applied studies to identify sources of resistance to physiological disorders (scald and drought), field diseases (fire blight and scab), and postharvest decay (blue mould and bitter rot; Janisiewicz *et al*., 2008; Jurick *et al*., 2011). Identification of resistance phenotypes was the first important step toward crafting a decay‐resistant commercial apple cultivar by conventional breeding, genetic modification, or a combination of the two. Without such foundational studies, the current state of knowledge regarding host resistance mechanisms in apple would not exist.

Both classical genetic and omics‐based studies have helped to discover genes and markers mediating resistance in wild apple fruit to postharvest decay that was originally discovered by Janisiewicz *et al*. (2008) and Jurick *et al*. (2011). Norelli *et al*. (2017) identified the quantitative trait locus (QTL) qM‐Pe3.1 on linkage group 3 for blue mould resistance in the mapping population GMAL4593 (Norelli *et al*., 2017). Ballester *et al*. (2017) then characterized the transcriptomic response of resistant apple fruit to wounding and inoculation with *P. expansum* using RNA‐Seq. Their transcriptomic analyses reinforced previous findings in that a higher basal level of resistance and a more rapid and intense defence response to wounding was pivotal for the resistance phenotype. Interestingly, 20 differentially expressed genes were mapped to the qM‐Pe3.1 QTL, which comprise candidate genes responsible for the resistance found in wild apple fruit. Translating these fundamental findings was recently achieved by Luo *et al*. (2020), who introgressed the qM‐Pe3.1 resistance allele via rapid cycle breeding using the transgenic line T1190 constitutively expressing the BpMADS4 early‐flowering gene (Luo *et al*., 2020). While this research moves the fundamental science forward, there is a lack of practical impact of these findings. Hence this research should be viewed as preliminary but shows proof of concept that a QTL can be moved from wild to commercial apples. Nevertheless, we are one step closer to producing commercial cultivars with resistance to blue mould.

## 6 FUTURE PERSPECTIVES

**FIGURE 2:**
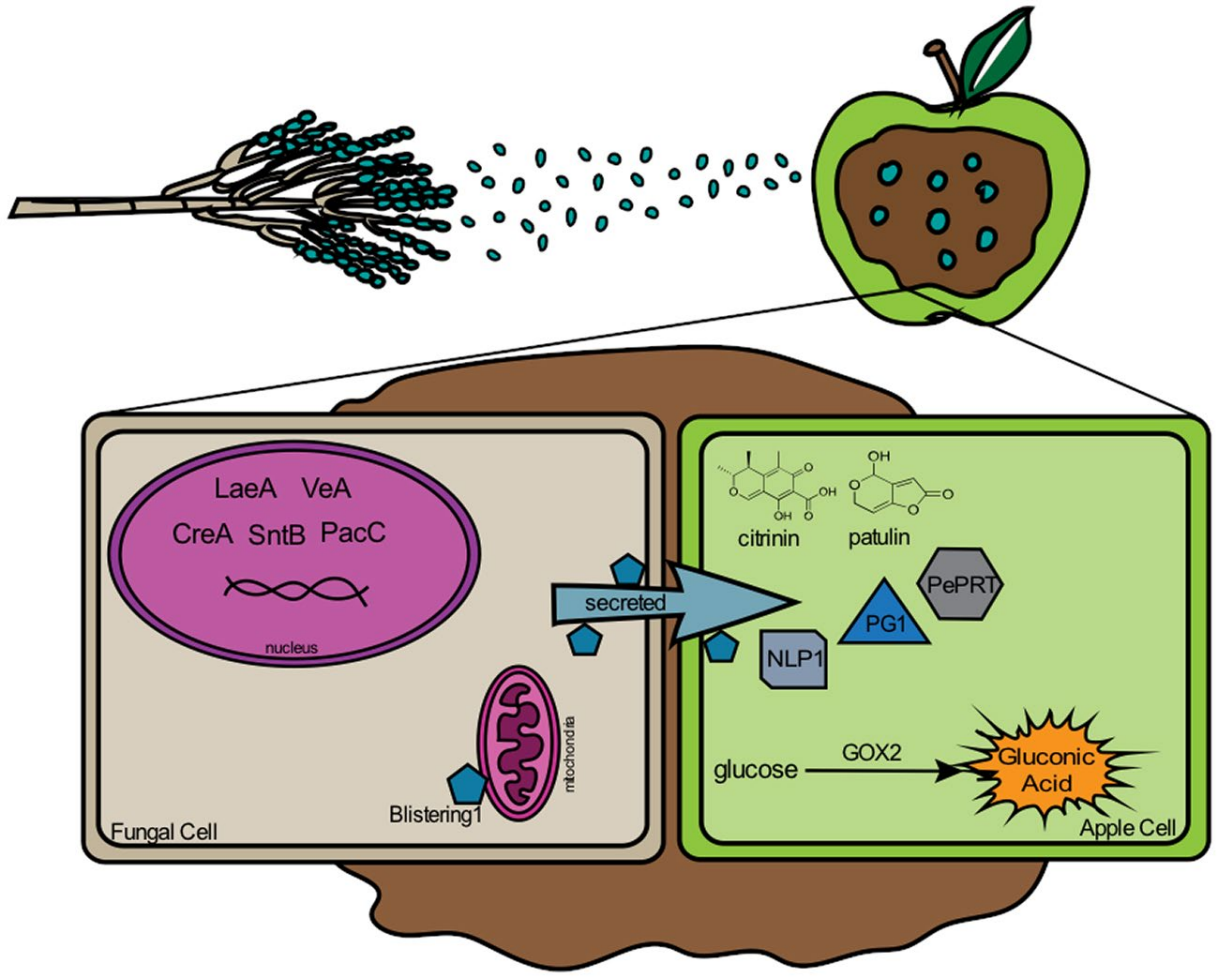
Blue mould disease model in apples. Conidia enter wounded tissue where they germinate and develop as hyphae. In the leading edge of the infection, PacC mediates pH regulation via up‐regulation of glucose oxidase, which is secreted and catalyses the conversion of glucose to gluconic acid, which mediates acidification of the host tissue. Effector proteins and cell wall‐degrading enzymes such as polygalacturonase 1 (PG1), NEP1‐like protein 1 (NLP1), and PePRT are secreted. Protein secretion is mediated by Blistering1, putatively localized to the fungal cell mitochondria. The mycotoxin secondary metabolites patulin and citrinin are secreted into apple tissue. Patulin regulation is mediated by global regulators LaeA, VeA, CreA, and SntB although signal transduction pathways relaying the message are still unknown

Great advances have been made towards obtaining a model that informs us how *P. expansum* causes disease in apples (Figure 2). However, most of this effort has been led by an interest in learning how patulin is regulated and what genes are important to incite decay. While this knowledge and findings have been of great value, there are many areas that need to be explored further. Some of the molecular aspects that we are yet to investigate are the signalling pathways and regulators governing decay. As a necrotrophic pathogen, another area to explore is the identification of effectors that enable *P. expansum* to have this lifestyle and their role, interaction, and function in the host tissue. These have the greatest potential to translate into novel control strategies as they could be blocked by a variety of different technologies to control decay. In addition, the current number of available *P. expansum* genomes could provide an opportunity to explore interspecific variation and the potential to develop multiple hypotheses on genes responsible for strain virulence and the role that potential sexual recombination plays in genome plasticity. Furthermore, by exploring additional fruit hosts, we could gain knowledge on conserved or unique mechanisms and their regulation, and strategies that govern host–pathogen specificity. Although it has been briefly explored, we can deepen our understanding on cultivar × strain virulence. In an ecological context, it would be interesting to understand the relevance and dynamics of microbial communities in the host on *P. expansum* colonization. This fundamental knowledge alone or in combination will advance the field not only in the possible development of novel and durable applications to control blue mould but also to contribute to the area of fundamental fungal molecular biology.

## Data Availability

Data sharing is not applicable to this article as no new data were created or analysed in this study.

## References

[mpp12990-bib-0001] Ali, N. and Degen, G.H. (2019) Citrinin biomarkers: a review of recent data and application to human exposure assessment. Archives of Toxicology, 93, 3057–3066. 31501918 10.1007/s00204-019-02570-y

[mpp12990-bib-0002] Alkan, N. , Espeso, E.A. and Prusky, D. (2013) Virulence regulation of phytopathogenic fungi by pH. Antioxidants & Redox Signaling, 19, 1012–1025. 23249178 10.1089/ars.2012.5062

[mpp12990-bib-0003] Andersen, B. , Smedsgaard, J. and Frisvad, J.C. (2004) *Penicillium expansum*: consistent production of patulin, chaetoglobosins, and other secondary metabolites in culture and their natural occurrence in fruit products. Journal of Agricultural and Food Chemistry, 52, 2421–2428. 15080656 10.1021/jf035406k

[mpp12990-bib-0004] Ballester, A.R. , Houben, M. , Levin, E. , Sela, N. , Lazaro, C.S. , Carmona, L. *et al* (2015) Genome, transcriptome, and functional analysis of *Penicillium expansum* provide new insights into secondary metabolism and pathogenicity. Molecular Plant‐Microbe Interactions, 28, 232–248. 25338147 10.1094/MPMI-09-14-0261-FI

[mpp12990-bib-0005] Ballester, A.R. , Norelli, J. , Burchard, E. , Abdelfattah, A. , Levin, E. , González‐Candelas, L. *et al* (2017). Transcriptomic response of resistant (PI613981‐*Malus sieversii*) and susceptible (“Royal Gala”) genotypes of apple to blue mold (*Penicillium expansum*) Infection. Frontiers in Plant Science, 8, s 1981.–. 10.3389/fpls.2017.01981 PMC5696741 29201037

[mpp12990-bib-0006] Barad, S. , Horowitz, S.B. , Moscovitz, O. , Lichter, A. , Sherman, A. and Prusky, D. (2012) A *Penicillium expansum* glucose oxidase‐encoding gene, GOX2, is essential for gluconic acid production and acidification during colonization of deciduous fruit. Molecular Plant‐Microbe Interactions, 25, 779–788. 22352719 10.1094/MPMI-01-12-0002

[mpp12990-bib-0007] Barad, S. , Horowitz, S.B. , Kobiler, I. , Sherman, A. and Prusky, D. (2014) Accumulation of the mycotoxin patulin in the presence of gluconic acid contributes to pathogenicity of *Penicillium expansum* . Molecular Plant‐Microbe Interactions, 27, 66–77. 24024763 10.1094/MPMI-05-13-0138-R

[mpp12990-bib-0008] Barad, S. , Espeso, E.A. , Sherman, A. and Prusky, D. (2016a) Ammonia activates pacC and patulin accumulation in an acidic environment during apple colonization by *Penicillium expansum* . Molecular Plant Pathology, 17, 727–740. 26420024 10.1111/mpp.12327 PMC6638319

[mpp12990-bib-0009] Barad, S. , Sela, N. , Kumar, D. , Kumar‐Dubey, A. , Glam‐Matana, N. , Sherman, A. *et al* (2016b) Fungal and host transcriptome analysis of pH‐regulated genes during colonization of apple fruits by *Penicillium expansum* . BMC Genomics, 17, 330. 27146851 10.1186/s12864-016-2665-7 PMC4855365

[mpp12990-bib-0010] Beattie, S.R. , Mark, K.M.K. , Thammahong, A. , Ries, L.N.A. , Dhingra, S. , Caffrey‐Carr, A.K. *et al* (2017) Filamentous fungal carbon catabolite repression supports metabolic plasticity and stress responses essential for disease progression. PLoS Pathogens, 13, e1006340. 28423062 10.1371/journal.ppat.1006340 PMC5411099

[mpp12990-bib-0011] Bok, J.W. and Keller, N.P. (2016) Insight into fungal secondary metabolism from ten years of LaeA research In: Hoffmeister D. (Ed.) Biochemistry and Molecular Biology. The Mycota (A Comprehensive Treatise on Fungi as Experimental Systems for Basic and Applied Research). Vol. III, Cham Springer, pp. 21–29.

[mpp12990-bib-0012] Chen, Y. , Li, B. , Xu, X. , Zhang, Z. and Tian, S. (2018) The pH‐responsive PacC transcription factor plays pivotal roles in virulence and patulin biosynthesis in *Penicillium expansum* . Environmental Microbiology, 20, 4063–4078. 30370586 10.1111/1462-2920.14453

[mpp12990-bib-0013] Ciegler, A. , Vesonder, R.F. and Jackson, L.K. (1977) Production and biological activity of patulin and citrinin from *Penicillium expansum* . Applied and Environmental Microbiology, 33, 1004–1006. 559470 10.1128/aem.33.4.1004-1006.1977 PMC170810

[mpp12990-bib-0014] Devaraj, H. , Suseela, R.E. and Devaraj, N. (1986) Patulin toxicosis in chicks. Current Science, 55, 998–999.

[mpp12990-bib-0015] Doughari, J.H. (2015) The occurrence, properties and significance of citrinin mycotoxin. Journal of Plant Pathology and Microbiology, 6, 11.

[mpp12990-bib-0016] El Hajj Assaf, C. , Snini, S.P. , Tadrist, S. , Bailly, S. , Naylies, C. , Oswald, I.P. *et al* (2018) Impact of *veA* on the development, aggressiveness, dissemination and secondary metabolism of *Penicillium expansum* . Molecular Plant Pathology, 19, 1971–1983. 10.1111/mpp.12673 PMC6638001 29517851

[mpp12990-bib-0017] Errampalli, D. (2004) Effect of fludioxonil on germination and growth of *Penicillium expansum* and decay in apple cvs. Empire and Gala. Crop Protection, 23, 811–817.

[mpp12990-bib-0018] Fasoyin, O.E. , Wang, B. , Qiu, M. , Han, X. , Chung, K.R. and Wang, S. (2018) Carbon catabolite repression gene creA regulates morphology, aflatoxin biosynthesis and virulence in *Aspergillus flavus* . Fungal Genetics and Biology, 115, 41–51. 29655909 10.1016/j.fgb.2018.04.008

[mpp12990-bib-0019] Feng, B. , Zhu, X. , Fu, L. , Lv, R. , Storey, D. , Tooley, P. *et al* (2014) Characterization of necrosis‐inducing NLP proteins in *Phytophthora capsici* . BMC Plant Biology, 14, 126. 24886309 10.1186/1471-2229-14-126 PMC4023171

[mpp12990-bib-0020] Forsline, P.L. , Aldwinkle, H. , Dickenson, E. , Luby, J. & Hokanson, S. (2003) Collection, maintenance and characterization of wild apples of central Asia. Horticultural Reviews, 29, 1–61.

[mpp12990-bib-0021] Hadas, Y. , Goldberg, I. , Pines, O. and Prusky, D. (2007) Involvement of gluconic acid and glucose oxidase in the pathogenicity of *Penicillium expansum* in apples. Phytopathology, 97, 384–390. 18943660 10.1094/PHYTO-97-3-0384

[mpp12990-bib-0022] He, Y. and Cox, R.J. (2016) The molecular steps of citrinin biosynthesis in fungi. Chemical Science, 7, 2119–2127. 29899939 10.1039/c5sc04027b PMC5968754

[mpp12990-bib-0023] Hokanson, S.C. , McFerson, J.R. , Forsline, P.L. , Lamboy, W.F. , Luby, J.J. , Djangaliev, A.D. *et al* (1997) Collecting and managing wild *Malus* germplasm in its center of diversity. HortScience, 32, 173–176.

[mpp12990-bib-0025] Janisiewicz, N.W. and Jeffers, S.N. (1997) Efficacy of commercial formulation of two biofungicides for control of blue mold and gray mold of apples in cold storage. Crop Protection, 16, 629–633.

[mpp12990-bib-0026] Janisiewicz, W.J. , Saftner, R.A. , Conway, W.S. and Forsline, P.L. (2008) Preliminary evaluation of apple germplasm from Kazakhstan for resistance to postharvest blue mold in fruit caused by *Penicillium expansum* . HortScience, 43, 420–426.

[mpp12990-bib-0027] de Jonge, R. , van Esse, H.P. , Kombrink, A. , Shinya, T. , Desaki, Y. , Bours, R. *et al* (2010) Conserved fungal LysM effector Ecp6 prevents chitin‐triggered immunity in plants. Science, 329, 953–955. 20724636 10.1126/science.1190859

[mpp12990-bib-0028] Julca, I. , Droby, S. , Sela, N. , Marcet‐Houben, M. and Gabaldón, T. (2015) Contrasting genomic diversity in two closely related postharvest pathogens: *Penicillium digitatum* and *Penicillium expansum* . Genome Biology and Evolution, 8, 218–227. 26672008 10.1093/gbe/evv252 PMC4758248

[mpp12990-bib-0029] Jurick II, W.M. , Janisiewicz, W.J. , Saftner, R.A. , Vico, I. , Gaskins, V.L. , Park, E. *et al* (2011) Identification of wild apple germplasm (*Malus* spp) accessions with resistance to the postharvest decay pathogens *Penicillium expansum* and *Colletotrichum acutatum* . Plant Breeding, 130, 481–486.

[mpp12990-bib-0030] Jurick, W. , Vico, I. , Gaskins, V. , Kari, P. , Eunhee, P. , Wojciech, J. *et al* (2012) Carbon, nitrogen and pH regulate the production and activity of a polygalacturonase isozyme produced by *Penicillium expansum* . Archives of Phytopathology and Plant Protection, 45, 1101–1114.

[mpp12990-bib-0086] Jurick II, W. , Macarisin, O. , Gaskins, V. , Janisiewicz, W. , Peter, K. and Cox, K. (2019) Baseline sensitivity of *Penicillium* spp. to difenoconazole. Plant Disease, 103, 331–337. 30562129 10.1094/PDIS-05-18-0860-RE

[mpp12990-bib-0031] Jurick II, W.M. , Peng, H. , Beard, H.S. , Garrett, W.M. , Lichtner, F.J. , Luciano‐Rosario, D. *et al* (2020) Blistering1 modulates *Penicillium expansum* virulence via vesicle‐mediated protein secretion. Molecular and Cell Proteomics, 19, 344–361. 10.1074/mcp.RA119.001831 PMC7000123 31871254

[mpp12990-bib-0032] Keller, N.P. (2017) Heterogeneity confounds establishment of “a” model microbial strain. mBio, 8, e00135–e217. 28223452 10.1128/mBio.00135-17 PMC5358909

[mpp12990-bib-0033] Khodadadi, F. , González, J.B. , Martin, P.L. , Giroux, E. , Bilodeau, G.J. , Peter, K.A. *et al* (2020) Identification and characterization of *Colletotrichum* species causing apple bitter rot in New York and description of *C. noveboracense* sp. nov. Scientific Reports, 10, 11043. 32632221 10.1038/s41598-020-66761-9 PMC7338416

[mpp12990-bib-0034] Kim, H.Y. , Heo, D.Y. , Park, H.M. , Singh, D. and Lee, C.H. (2016) Metabolomic and transcriptomic comparison of solid‐state and submerged fermentation of *Penicillium expansum* KACC 40815. PLoS One, 11, e0149012. 26863302 10.1371/journal.pone.0149012 PMC4749308

[mpp12990-bib-0035] Kleemann, J. , Rincon‐Rivera, L.J. , Takahara, H. , Neumann, U. , van Themaat, E.V.L. , van der Does, H.C. *et al* (2012) Sequential delivery of host‐induced virulence effectors by appressoria and intracellular hyphae of the phytopathogen *Colletotrichum higginsianum* . PLoS Pathogens, 8, e1002643. 22496661 10.1371/journal.ppat.1002643 PMC3320591

[mpp12990-bib-0036] Kombrink, A. and Thomma, B.P. (2013) LysM effectors: secreted proteins supporting fungal life. PLoS Pathogens, 9, e1003769. 24348247 10.1371/journal.ppat.1003769 PMC3861536

[mpp12990-bib-0037] Kumar, D. , Barad, S. , Chen, Y. , Luo, X. , Tannous, J. , Dubey, A. *et al* (2016) LaeA regulation of secondary metabolism modulates virulence in *Penicillium expansum* and is mediated by sucrose. Molecular Plant Pathology, 18, 1150–1163. 27528575 10.1111/mpp.12469 PMC6638289

[mpp12990-bib-0038] Levin, E. , Ballester, A.R. , Raphael, G. , Feigenberg, O. , Liu, Y. , Norelli, J. *et al* (2017) Identification and characterization of LysM effectors in *Penicillium expansum* . PLoS One, 12, e0186023. 29084256 10.1371/journal.pone.0186023 PMC5662087

[mpp12990-bib-0039] Levin, E. , Raphael, G. , Ma, J. , Ballester, A.R. , Feygenberg, O. , Norelli, J. *et al* (2019) Identification and functional analysis of NLP‐encoding genes from the postharvest pathogen *Penicillium expansum* . Microorganisms, 7, 175. 10.3390/microorganisms7060175 PMC6616513 31208074

[mpp12990-bib-0085] Li, T. , Jiang, G. , Qu, H. , *et al.* (2017) Comparative transcriptome analysis of *Penicillium citrinum* cultured with different carbon sources identifies genes involved in citrinin biosynthesis. Toxins, 9, 69. 10.3390/toxins9020069 PMC5331448 28230802

[mpp12990-bib-0040] Li, H.X. and Xiao, C.L. (2008) Characterization of fludioxonil‐resistant and pyrimethanil‐resistant phenotypes of *Penicillium expansum* from apple. Phytopathology, 98, 427–435. 18944191 10.1094/PHYTO-98-4-0427

[mpp12990-bib-0042] Li, B. , Zong, Y. , Du, Z. , Chen, Y. , Zhang, Z. , Qin, G. *et al* (2015) Genomic characterization reveals insights into patulin biosynthesis and pathogenicity in *Penicillium* species. Molecular Plant‐Microbe Interactions, 28, 635–647. 25625822 10.1094/MPMI-12-14-0398-FI

[mpp12990-bib-0041] Li, B. , Chen, Y. , Zong, Y. , Shang, Y. , Zhang, Z. , Wang, X. *et al* (2019) Dissection of patulin biosynthesis, spatial control and regulation mechanism in *Penicillium expansum* . Environmental Microbiology, 21, 1124–1139. 30680886 10.1111/1462-2920.14542

[mpp12990-bib-0024] Loi, D.J. , Zhou, T. , Tsao, R. and Marcone, M.F. (2017) Mitigation of patulin in fresh and processed foods and beverages. Toxins, 9, 157. 10.3390/toxins9050157 PMC5450705 28492465

[mpp12990-bib-0043] Luo, Z. , Qin, Y. , Pei, Y. and Keyhani, N.O. (2014) Ablation of the creA regulator results in amino acid toxicity, temperature sensitivity, pleiotropic effects on cellular development and loss of virulence in the filamentous fungus *Beauveria bassiana* . Environmental Microbiology, 16, 1122–1136. 24320078 10.1111/1462-2920.12352

[mpp12990-bib-0044] Luo, F. , Evans, K. , Norelli, J.L. , Zhang, Z. and Peace, C. (2020) Prospects for achieving durable disease resistance with elite fruit quality in apple breeding. Tree Genetics & Genomes, 16, 21.

[mpp12990-bib-0045] Malandrakis, A.A. , Vattis, K.N. , Markoglou, A.N. and Karaoglanidis, G.S. (2017) Characterization of boscalid‐resistance conferring mutations in the SdhB subunit of respiratory complex II and impact on fitness and mycotoxin production in *Penicillium expansum* laboratory strains. Pesticide Biochemistry and Physiology, 133, 97–103. 10.1016/j.pestbp.2017.03.009 28456312

[mpp12990-bib-0046] Norelli, J.L. , Wisniewski, M. , Fazio, G. , Burchard, E. , Gutierrez, B. , Levin, E. *et al* (2017) Genotyping‐by‐sequencing markers facilitate the identification of quantitative trait loci controlling resistance to *Penicillium expansum* in *Malus sieversii* . PLoS One, 12, e0172949. 28257442 10.1371/journal.pone.0172949 PMC5336245

[mpp12990-bib-0047] de Oliveira Filho, J.W.G. , Islam, M.T. , Ali, E.S. , Uddin, S.J. , Santos, J.V.O. , de Alencar, M.V.O.B. *et al* (2017) A comprehensive review on biological properties of citrinin. Food and Chemical Toxicology, 110, 130–141. 28993214 10.1016/j.fct.2017.10.002

[mpp12990-bib-0048] Pfannenstiel, B.T. , Zhao, X. , Wortman, J. , Wiemman, P. , Throckmorton, K. , Spraker, J.E. *et al* (2017) Revitalization of a forward genetic screen identifies three new regulators of fungal secondary metabolism in the genus Aspergillus. mBio, 8, e01246–e1317. 28874473 10.1128/mBio.01246-17 PMC5587912

[mpp12990-bib-0049] Pfannenstiel, B.T. , Greco, C. , Sukowaty, A.T. and Keller, N.P. (2018) The epigenetic reader SntB regulates secondary metabolism, development and global histone modifications in *Aspergillus flavus* . Fungal Genetics and Biology, 120, 9–18. 30130575 10.1016/j.fgb.2018.08.004 PMC6215504

[mpp12990-bib-0050] Pianzzola, M.J. , Moscatelli, M. and Vero, S. (2004) Characterization of *Penicillium* isolates associated with blue mold on apple in Uruguay. Plant Disease, 88, 23–28. 30812451 10.1094/PDIS.2004.88.1.23

[mpp12990-bib-0051] Prusky, D. , McEvoy, J.L. , Saftner, R. , Conway, W.S. and Jones, R. (2004) Relationship between host acidification and virulence of *Penicillium* spp. on apple and citrus fruit. Phytopathology, 94, 44–51. 18943818 10.1094/PHYTO.2004.94.1.44

[mpp12990-bib-0052] Puel, O. , Galtier, P. and Oswald, I.P. (2010) Biosynthesis and toxological effects of patulin. Toxins, 2, 613. 22069602 10.3390/toxins2040613 PMC3153204

[mpp12990-bib-0053] Rice, S.L. , Beuchat, L.R. and Worthington, R.E. (1977) Patulin production by *Byssochlamys* spp. in fruit juices. Applied and Environmental Microbiology, 34, 791–796. 596876 10.1128/aem.34.6.791-796.1977 PMC242749

[mpp12990-bib-0054] Rosenberger, D.A. (1990) Blue mold In: Jones A.L. and Aldwinkle H.S. (Eds.) Compendium of Apple and Pear Diseases. St Paul, MN: APS Press, pp. 54–55.

[mpp12990-bib-0055] Rosenberger, D.A. (2012) Sanitize apple storage rooms to minimize postharvest decays. Scaffolds Fruit Journal, 21, 4–5.

[mpp12990-bib-0056] Rosenberger, D.A. , Engle, C.A. , Meyer, F.W. and Watkins, C.B. (2006) *Penicillium expansum* invades apples through stems during controlled atmosphere storage. Plant Health Progress, 7 10.1094/PHP-2006-1213-01-R

[mpp12990-bib-0057] Russell, P.E. (2004) Sensitivity baselines in fungicide resistance research and management. FRAC Monograph, 3, 1–54.

[mpp12990-bib-0058] Saleh, I. and Goktepe, I. (2019) The characteristics, occurrence, and toxicological effects of patulin. Food and Chemical Toxicology, 129, 301–311. 31029720 10.1016/j.fct.2019.04.036

[mpp12990-bib-0059] Sanderson, P.G. and Spotts, R.A. (1995) Postharvest decay of winter pear and apple fruit caused by species of *Penicillium* . Phytopathology, 85, 103–110.

[mpp12990-bib-0060] Sanzani, S.M. , Reverberi, M. , Punelli, M. , Ippolito, A. and Fanelli, C. (2012) Study on the role of patulin on pathogenicity and virulence of *Penicillium expansum* . International Journal of Food Microbiology, 153, 323–331. 22189024 10.1016/j.ijfoodmicro.2011.11.021

[mpp12990-bib-0061] Sarikaya‐Bayram, Ö. , Palmer, J.M. , Keller, N. , Braus, G.H. and Bayram, Ö. (2015) One Juliet and four Romeos: VeA and its methyltransferases. Frontiers in Microbiology, 6, 1. 25653648 10.3389/fmicb.2015.00001 PMC4299510

[mpp12990-bib-0062] Selvig, K. and Alspaugh, J.A. (2011) pH response pathways in fungi: adapting to host‐derived and environmental signals. Microbiology, 39, 249–256. 10.5941/MYCO.2011.39.4.249 PMC3385132 22783112

[mpp12990-bib-0063] Sholberg, P.L. and Haag, P.D. (1996) Incidence of postharvest pathogens of stored apples in British Columbia. Canadian Journal of Plant Pathology, 18, 81–85.

[mpp12990-bib-0064] Snini, S.P. , Tannous, J. , Heuillard, P. , Bailly, S. , Lippi, Y. , Zehraoui, E. *et al* (2016) Patulin is a cultivar‐dependent aggressiveness factor favouring the colonization of apples by *Penicillium expansum* . Molecular Plant Pathology, 17, 920–930. 26582186 10.1111/mpp.12338 PMC6638343

[mpp12990-bib-0065] Spotts, R.A. , Cervantes, L.A. and Mielke, E.A. (1999) Variability in postharvest decay among apple cultivars. Plant Disease, 83, 1051–1054. 30841275 10.1094/PDIS.1999.83.11.1051

[mpp12990-bib-0087] Spotts, R. A. and Cervantes, L. A. (1994) Contamination of harvest binds with pear decay fungi and evaluation of disinfestants on plastic and wood bin material. Acta Horticulturae, 367, 419–425.

[mpp12990-bib-0066] Sutton, T.B. , Aldwinckle, A. , Agnello, A.M. and Walgenbach, J.F. (2014) Compendium of Apple and Pear Diseases and Pests, 2nd edition. St Paul, MN American Phytopathological Society.

[mpp12990-bib-0067] Tannous, J. , El Khoury, R. , Snini, S.P. , Lippi, Y. , El Khoury, A. , Atoui, A. *et al* (2014) Sequencing, physical organization and kinetic expression of the patulin biosynthetic gene cluster from *Penicillium expansum* . International Journal of Food Microbiology, 189, 51–60. 25120234 10.1016/j.ijfoodmicro.2014.07.028

[mpp12990-bib-0068] Tannous, J. , Keller, N.P. , Atoui, A. , El Khoury, A. , Lteif, R. , Oswald, I.P. *et al* (2018a) Secondary metabolism in *Penicillium expansum*: Emphasis on recent advances in patulin research. Critical Reviews in Food Science and Nutrition, 58, 2082–2098. 28362209 10.1080/10408398.2017.1305945

[mpp12990-bib-0069] Tannous, J. , Kumar, D. , Sela, N. , Sionov, E. , Prusky, D. and Keller, N.P. (2018b) Fungal attack and host defence pathways unveiled in near‐avirulent interactions of *Penicillium expansum* creA mutants on apples. Molecular Plant Pathology, 19, 2635–2650. 30047230 10.1111/mpp.12734 PMC6638163

[mpp12990-bib-0070] Tannous, J. , Barda, O. , Luciano‐Rosario, D. , Prusky, D.B. , Sionov, E. and Keller, N.P. (2020) New insight into pathogenicity and secondary metabolism of the plant pathogen *Penicillium expansum* through deletion of the epigenetic reader SntB. Frontiers in Microbiology, 11, 610. 32328048 10.3389/fmicb.2020.00610 PMC7160234

[mpp12990-bib-0071] Touhami, N. , Soukup, S.T. , Schmidt‐Heydt, M. , Kulling, S.E. and Geisen, R. (2018) Citrinin as an accessory establishment factor of *Penicillium expansum* for the colonization of apples. International Journal of Food Microbiology, 266, 224–233. 29268208 10.1016/j.ijfoodmicro.2017.12.007

[mpp12990-bib-0072] Vidal, A. , Ouhibi, S. , Ghali, R. , Hedhili, A. , De Saeger, S. and De Boevre, M. (2019) The mycotoxin patulin: an updated short review on occurrence, toxicity and analytical challenges. Food and Chemical Toxicology, 129, 249–256. 31042591 10.1016/j.fct.2019.04.048

[mpp12990-bib-0073] Wang, Y. , Feng, K. , Yang, H. , Zhang, Z. , Yuan, Y. and Yue, T. (2018) Effect of cinnamaldehyde and citral combination on transcriptional profile, growth, oxidative damage and patulin biosynthesis of *Penicillium expansum* . Frontiers in Microbiology, 9, 597. 29651282 10.3389/fmicb.2018.00597 PMC5884930

[mpp12990-bib-0074] Wang, K. , Zheng, X. , Zhang, X. , Zhao, L. , Yang, Q. , Boateng, N. *et al* (2019) Comparative transcriptomic analysis of the interaction between *Penicillium expansum* and apple fruit (*Malus pumila* Mill.) during early stages of infection. Microorganisms, 7, 495. 10.3390/microorganisms7110495 PMC6920851 31661784

[mpp12990-bib-0075] Wenneker, M. and Thomma, B.P.H.J. (2020) Latent postharvest pathogens of pome fruit and their management: from single measures to a systems intervention approach. European Journal of Plant Pathology, 156, 663–681.

[mpp12990-bib-0076] Wu, G. , Jurick II, W.M. , Lichtner, F.J. , Peng, H. , Yin, G. , Gaskins, V.L. *et al* (2019) Whole‐genome comparisons of *Penicillium* spp. reveals secondary metabolic gene clusters and candidate genes associated with fungal aggressiveness during apple fruit decay. PeerJ, 7, e6170. 30643697 10.7717/peerj.6170 PMC6330040

[mpp12990-bib-0077] Xiao, C.L. and Boal, R.J. (2009) Residual activity of fludioxonil and pyrimethanil against *Penicillium expansum* on apple fruit. Plant Disease, 93, 1003–1008. 30754370 10.1094/PDIS-93-10-1003

[mpp12990-bib-0078] Xu, B. , Jia, X. , Gu, L. and Sung, C. (2006) Review on the qualitative and quantitative analysis of the mycotoxin citrinin. Food Control, 17, 271–285.

[mpp12990-bib-0079] Yang, Y. , Zhao, H. , Barrero, R.A. , Zhang, B. , Sun, G. , Wilson, I.W. *et al* (2014) Genome sequencing and analysis of the paclitaxel‐producing endophytic fungus *Penicillium aurantiogriseum* NRRL 62431. BMC Genomics, 15, 69. 24460898 10.1186/1471-2164-15-69 PMC3925984

[mpp12990-bib-0080] Yao, C. , Conway, W.S. and Sams, C.E. (1996) Purification and characterization of a polygalacturonase produced by *Penicillium expansum* in apple fruit. Phytopathology, 86, 1160–1166.

[mpp12990-bib-0081] Yu, J. , Jurick II, W.M. , Cao, H. , Yin, Y. , Gaskins, V.L. , Losada, L. *et al* (2014) Draft genome sequence of *Penicillium expansum* strain R19, which causes postharvest decay of apple fruit. Genome Announcements, 2, e00635–e714. 24948776 10.1128/genomeA.00635-14 PMC4064041

[mpp12990-bib-0082] Zhong, L. , Carere, J. , Lu, Z. , Lu, F. and Zhou, T. (2018) Patulin in apples and apple‐based food products: the burdens and the mitigation strategies. Toxins, 10, 475. 10.3390/toxins10110475 PMC6267208 30445713

[mpp12990-bib-0083] Zhou, T. , Wang, X. , Luo, J. , Ye, B. , Zhou, Y. , Zhou, L. *et al* (2018a) Identification of differentially expressed genes involved in spore germination of *Penicillium expansum* by comparative transcriptome and proteome approaches. Microbiology Open, 7, e00562. 29205951 10.1002/mbo3.562 PMC6011939

[mpp12990-bib-0084] Zhou, T. , Wang, X. , Ye, B. , Shi, L. , Bai, X. and Lai, T. (2018b) Effects of essential oil decanal on growth and transcriptome of the postharvest fungal pathogen *Penicillium expansum* . Postharvest Biology and Technology, 145, 203–212.

